# What Types of Happiness Do Korean Adults Pursue?—Comparison of Seven Happiness Types

**DOI:** 10.3390/ijerph17051502

**Published:** 2020-02-26

**Authors:** Young Yim Doh, Ji-Bum Chung

**Affiliations:** 1 Graduate School of Culture Technology, Korea Advanced Institute of Science and Technology, Daejeon 34141, Korea; yydoh@kaist.ac.kr; 2 School of Urban and Environmental Engineering, Ulsan National Institute of Science and Technology, Ulsan 44919, Korea

**Keywords:** Korean adult, types of happiness, subjectivity, Q methodology, mixed-method approach

## Abstract

Although Korea has achieved successful economic, social, cultural, and technological development over the past decades, Korean people do not seem to be particularly happy. To enhance an individual’s happiness, we need to be aware of what situations and environmental conditions are conducive for happiness and explore the values of happiness we pursue. This study investigated the types of happiness expressed by Korean people using a mixed-method approach. Personal in-depth (*n* = 15) and focus group (*n* = 16) interviews were conducted with people who reported feeling a high level of happiness. Happiness categorization was conducted using Q methodology (*n* = 63). Subsequently, we surveyed 999 nationally representative samples of Korean adults to generalize the results of the Q analysis. The findings revealed seven types of adult happiness in Korea: (1) Self-actualization, (2) Belongingness, (3) Mission, (4) Social recognition, (5) Enjoyment, (6) Material success, and (7) Parenting. The combined results of the qualitative and quantitative analyses showed that in Korea, people pursuing money or social success feel the unhappiest, whereas people pursuing a mission or sense of belonging feel the happiest. In conclusion, we discussed the need for happiness literacy education, to provide each adult an opportunity to understand the type of happiness they pursue.

## 1. Introduction

Although Korea has achieved successful economic, social, cultural, and technological development over the past decades, Korean people do not seem to be particularly happy. According to the World Bank [1], the GDP per capita (PPP) of the Republic of Korea in 2018 was over USD 40,000, ranking it 31st among 182 in the world (17%). However, a recent survey by the World Happiness Report [2] revealed that Koreans’ happiness index is 5.895 out of 10, ranking it 54th in the world, markedly lower than other countries with lower GDP levels. In particular, “Be Rich” was a kind of declarative slogan that represented the life goal for Korean people after the Korean War of the 1950s. Many Koreans still believe that material success is an absolute indicator of a happy life. As an adult, we usually do not have the opportunity to learn about happiness in a formal education environment and our understanding of happiness comes from informal sources, such as parents, friends, life experiences, and the mass media [3]. When we do not know what type of happiness we really want, we easily follow the standards of happiness set by others or the social norms of society. To enhance an individual’s happiness, we need to be aware of the situations and environmental conditions that make us feel happy and to explore the values of happiness we pursue [4,5].

Happiness is fundamentally a subjective feeling. How happy one feels can vary widely from person to person. As de Guzman et al. [6] mentioned, the definition of happiness can “differ from person to person, and varies among cultures, economic status, social connectedness, spiritual upbringing and daily situations” (p. 913). Recently, researchers reached the consensus that happiness studies need qualitative, culturally sensitive, and subjective approaches, as well as objective and statistical approaches [7,8,9]. Several studies have sought to identify the diverse conceptions of happiness in various regions through qualitative or mixed methodology [10,11,12]. Although an individual’s average level of happiness and the variance in adult happiness is strongly influenced by genetic factors and by the effects of experiences on each individual [13,14], the recent findings suggest that the various concepts of adult happiness are created by complex socio-environmental structures, such as cultural values, social roles, and life cycles [10,15,16,17,18,19].

Korea is located in a unique position, both geopolitically and historically. It is connected to China on one side and to Japan on the other. It has also been influenced by Western countries, such as the United States, because of the division of South and North Korea. In addition, the nation-wide diffusion of Internet networks and the rapid acceptance of technology allowed for the integration of a range of happiness values from both the East and the West. All these socio-economic conditions have caused a differentiation in happiness concepts among Korean people. An early study of the structure of “a happy life” among Korean adults [20] identified 16 factors, categorized in three groups, that contribute to the happiness of Korean adults: Intrapersonal (self-acceptance, personal growth, autonomy, positive attitude, and religion), Interpersonal (helping others, relationship with children, relationship with parents and siblings, intimate relationship, and relationship with others), and Living conditions (money, leisure, social status, social environment, appearance, and health). Based on these results, the authors argued that the concept of happiness in Korean adults included more than life satisfaction or positive affect. Psychological functioning, other’s respect, relationships, and money are important sources of happiness in Korean adults [20]. A more recent study compared the concept of happiness among American college students (emotional, psychological, and social factors), with the concept of happiness among Korean college students, where the former categories were further subdivided into emotional, psychological, relational, social, and socioeconomic factors. Family relations and socioeconomic factors (economic power, religion, appearance, health, leisure, etc.) in particular were found to be important for Koreans’ concept of happiness [21].

Previous studies have a tendency towards a variable centric approach to finding out what factors contributed to Korean adult happiness. A variable centric approach seeks to identify happiness variables in a pool of individuals and then to test if there is any correlation between the variables. However, to promote individual happiness, it is better to take a person-centric approach [22]. A person-centric approach seeks to test whether subgroups of individuals can be distinguished by their profile of happiness dimensions, which can be mapped to a “type.” In other words, it is necessary to deeply understand the different contexts in which individuals are involved and to devise appropriate intervention strategies. To do this, it is necessary to explore ways to measure the subjectivity of happiness, including the context of the individual’s life, what features are characteristic of each type, and how happiness differs in each type. For example, Kim [23] conducted a Q analysis on 38 health-related university students in Gyeongbuk-province, Korea and identified three types of happiness: comfort, self-contentment, and acknowledgment. Ryu [24] identified four types of happiness in 41 kindergarten teachers in Korea: self-realization, inner satisfaction, social relationships, and personal belief. A research conducted by Shim [9] attempted to identify the subjectivity of happiness in 40 undergraduate students in Korea. The results proposed four types of happiness: a self-directed type, an oriental and wealth-seeking type, a realistic and pleasure-seeking type, and an altruistic and relationship-seeking type.

Until now, almost all Q-methods done to classify happiness in Korean adults have used purposively selected small groups of people with a limited age range in special localized settings. Although they gave us the important insight that happiness varies widely from person to person, the models they presented were too simple and lacked the systematic validation of a larger cohort. In the present study, we used a mixed-method approach to define peoples’ diverse happiness conception types qualitatively and then to verify them quantitatively. We pursued the development and verification of a model for Korean adults’ happiness type using a mixed-method approach combining the Q methodology and the survey method. Our focus was on the following central research questions. First, how can we subdivide subjective types of Korean adults’ happiness? Second, how can we describe the behavioral characteristics of a happy and an unhappy life perceived by each type? Third, how can we verify and generalize the subjective types of happiness statistically? Finally, how does each type differ with respect to the perception of household income and overall happiness?

## 2. Materials and Methods

We used an exploratory sequential mixed-method design approach in this study [25]. As the feeling of happiness can be very different from one person to another, we applied a Q methodology for a small purposive sample to explore patterns of individual happiness. Then, we conducted a survey of a nationally representative sample of Korean adults to generalize the previous results. All participants in the data-gathering steps (in-depth interview, focus group interview, Q sorting, and survey) were informed of the principle of anonymity and voluntary participation.

### 2.1. Q Methodology

Q methodology is a systematic means to examine personal experiences. It provides effective tools to investigate patterns in individual subjectivity, viewpoints, opinions, beliefs, attitudes, and self-perception [26]. For the feeling of individual happiness, Q methodology can be a useful tool to determine how happiness is subjectively perceived. From the viewpoint of statistical methodologies, “Q is a standard factor analysis turned on its side, with correlations computed between persons across a set of statements, rather than a standard “R method” correlation between traits (such as ratings of statements) across a set of persons” [27]. Q methodology uses Q and P samples. Q samples are statements or representations of communication contexts, whereas P samples refer to the persons who state their personal experiences and perform the Q sort.

### 2.2. Q Sampling

To construct statements of happiness for Q sampling, we conducted personal in-depth (*n* = 15) and focus group (*n* = 16) interviews (age range 29–56). Each of the 31 participants had diverse lifestyles. They were mostly in the middle-income group and had diverse occupations. They also reported feelings of a relatively high level of happiness. The reason we selected the interviewees who felt happy was that people who are happy find it easier to account for their state of happiness. We selected participants using a purposive and snowball sampling method. 

In-depth interviews and focus group interviews were conducted in semi-structured interviews in which open questions were asked to identify the characteristics that make up subjective happiness in each area of life. Deep questions were asked based on the participants’ responses. Ten detailed themes emerged: (1) Family Background; (2) Career/Job/Life Goals/Self-Development; (3) Community Engagement/Social Service; (4) Economic Resources/Income/Consumption; (5) Physical Health/Self-Management; (6) Human Relationships (Friends/Lovers/Spouse/Parents/Children); (7) Hobbies/Leisure/Cultural Life; (8) Religion/Future Preparation/Aging and Attitudes toward Death; (9) Residential Environments and Social Safety; and (10) Coping Styles for Stress.

Based on the results of the individual in-depth interviews, focus group interviews, and reviews of recent literature, we constructed a list of statements characterizing adult happiness in Korea. We gathered a total of 752 statements through the first Q sampling process. These statements were categorized and reduced to a smaller number of more meaningful statements. Subsequently, the appropriateness of the statements as Q statements was cross-evaluated by three interviewees who participated in in-depth interviews, and three academic experts in Q sampling. Finally, 93 statements were selected as Q statements (see Appendix A).

### 2.3. P Sampling

To determine the types of happiness in Korea, we purposively selected 63 adults (female: 50.5%) with various lifestyles to participate in this research. The participants’ ages fell within a wide range (20 s, 19.1%; 30 s, 21.1%; 40 s, 22.3%; 50 s, 17.9%; 60 s or older, 19.6%). The regions in which they lived were widely dispersed as well, including Seoul City and many rural areas. To obtain a complete picture of Korean adults, the participants selected were from a wide range of income groups (from 0 to 7000 USD per month) and held different types of jobs, including office workers, restaurant owners, writers, and cartoonists.

### 2.4. Q Sorting

The 63 participants (P sample) were asked to sort the 93 Q sample index cards (13 × 7.5 cm) on which each Q statement was written. The participants read the cards and then divided them into three groups: a “happy” (+) group if the statement described their status of happiness very well; an “unhappy” (–) group, if the statement described their status of unhappiness, and a “neutral” (0) group, if the statement was ambiguous. Then, the participants were asked to arrange the cards by the strength of happiness (unhappiness) on the Q sort table, a nine-point Likert scale table. We conducted the principal component factor analysis using the QUANL program for PC [28].

From the results of the analysis, we confirmed that three-, four-, six-, and seven-factor structures were statistically significant. After a comparison of the four models, we chose the seven-factor model based on factor structure diversity and factor loading scores. Factor loading scores presented a clear distinction between the factors. In Q methodology, factors refer to people. Therefore, we described it as “type.”

### 2.5. Survey Methods and Analysis

We developed the questionnaire for the survey of a happiness type scale using the results from the initial qualitative data collection and the Q methodology analysis (Table 1). The questionnaire item for perceived household income is “If you rank the Korean household income from 1 to 10, what do you think your household income level is?” (1, the lowest; 10, the highest; mean = 5.34; *SD* = 1.51; no one answered 10, so the maximum value was 9). We also asked the respondents to write down their estimated actual monthly income before taxes for their household in KRW 10,000 (KRW 10,000 is about USD 10; response rate = 67.4%; mean = 246.88; *SD* = 133.70). The questionnaire item for perceived overall happiness level is “Taken altogether, how happy would you say you are?” (1, not happy at all; 10, very happy; mean = 6.62; *SD* = 1.66).

Using these items, including demographic variables (age, sex, education level, marital status, number of children), a survey of 999 Korean adults aged 19 years and older, was conducted. A multi-stage stratified sampling method was used, referencing regions (excluding Jeju Island), ages, and sexes based on the nationwide population distribution (as of December 2010). The sampling error was ± 3.1% at a 95% confidence level (CI). The face-to-face interview survey was conducted from October 4 to 18, 2011, using a structured questionnaire.

### 2.6. Happiness Type Scale Creation Using Q Statements

In the present study, we used the scale creation method proposed by Danielson [27] to transform Q statements into survey questionnaire items. This method is a way of making the Likert-scale questionnaire items by reusing the 93 Q statements that characterize the happiness and unhappiness of Korean adults. The Q statements that had a z-score (weighted average of the ranking of Q statements within each factor [29] greater than or equal to 1 (z-score ≥ 1; meaning, happy; Likert scales for happiness {“H” in Table 1}) or less than or equal to −1 (z-score ≤ −1; meaning, unhappy; Likert scale for unhappiness {“U” in Table 1}) were used for the Likert-scale survey questionnaire items. 

### 2.7. Analyzing and Identifying Happiness Types

From the results of the survey, each respondent could be identified as being one of seven happiness types. The procedure for determining happiness types was as follows.

1. Create the seven Happiness Type Indexes T_1_, T_2_, T_3_, T_4_, T_5_, T_6_, and T_7_ that represent each type of happiness.
2. Multiply the response ratings for happiness and unhappiness (*x*_n_) and its z-score (*z*_n_) in each happiness type and add all the values. In this calculation, we only used the z-scores where the absolute value was greater than or equal to 1 (see Appendix A). To standardize the calculation, we divided the added values by the sum of z-scores of each type. The example formula for calculating the value of the Happiness Type Index of T1 is as follows.
  (1) $$T_{1}=\mathrm{Happiness}\;\mathrm{Score}\;+\;\mathrm{Unhappiness}\;\mathrm{Score}=\;\frac{x_{6}z_{6}+x_{8}z_{8}+x_{9}z_{9}+\ldots +x_{90}z_{90}}{z_{6}+z_{8}+z_{9}+\ldots +z_{90}}+\frac{x_{3}z_{3}+x_{12}z_{12}+x_{20}z_{20}+\ldots +x_{93}z_{93}}{z_{3}+z_{12}+z_{20}+\ldots +z_{93}}$$

The largest value among T_1_, T_2_, T_3_, T_4_, T_5_, T_6_, and T_7_ obtained by the above calculations was selected as the happiness type for each respondent.

## 3. Results

### 3.1. Seven Types of Happiness: Results of Q Analysis

From the results of the factor analysis, seven types of happiness were identified. The eigenvalues for the seven types were as follows: Factor 1 = 28.47, Factor 2 = 3.95, Factor 3 = 2.04, Factor 4 = 1.88, Factor 5 = 1.59, Factor 6 = 1.56, and Factor 7 = 1.50. These factors accounted for 65.05% of the total variance (see Table 1). The seven types of happiness were classified into the following names based on the life values pursued by each individual: Self-actualization, Belongingness, Mission, Social recognition, Enjoyment, Material success, and Parenting.

### 3.2. Happy and Unhappy Lives: Seven Types of Happiness

We confirmed the subjectivity of each group by describing, interpreting, and discussing the representative statements at an interpretation workshop where participants (*n* = 5), researchers (*n* = 3), and expert advisors (*n* = 2) were present. The workshop aimed to minimize the biases of researchers on the subjectivity analysis and to promote the validity and rigor of the interpretation through consensus among people with different perspectives and interests. The following descriptions of behavior patterns were gleaned from the workshop and provide the meaning for a happy or unhappy life for the seven types of happiness.

- *Self-actualization type*. Individuals who seek self-actualization for happiness, or the self-actualization type of person, are happy when they get energy from creative work that realizes their self-worth. They seek to experience professionalism, construct a distinct identity, and pursue a balanced life between work, home, hobby, and community participation. Meanwhile, they feel helpless when they are being manipulated by social recognition or money. They feel easily frustrated in a constrained environment where they cannot realize their dreams.
- *Belongingness type*. The belongingness type is happy when they feel a sense of belonging and live in a community among people with shared values. They willingly help others who are facing hardships. They are conforming optimists who are self-sufficient and find satisfaction in the small things in life. Meanwhile, if they cannot get along with community members because of shyness or the community they want to belong to rejects them, they are unhappy. As they have horizontal values, they cannot stand the pressure in hierarchical, organizational life.
- *Mission type*. The mission type is happy when they are able to carry out their life’s mission. They live an exemplary life with a sense of calling. This type enjoys the feeling of having a high level of control over a situation. They are pleased when they are able to overcome difficulties and fulfill their responsibilities in life. They are unhappy when the purpose of their life is unclear. They are confused when conditioned by material values instead of following their calling. This type feels helpless and deprived when they are in a situation where they cannot exercise control or when they have to depend on others.
- *Social recognition type*. The social recognition type is happy when they are able to obtain a high level of social status and are acknowledged by others. They are goal-driven and pleased when they are able to accomplish anything that requires a degree of effort. Meanwhile, it is difficult for them to endure conflict situations because they are overly conscious of social acceptance. As the image seen by others is important to them, it is not easy for them to reveal their true selves. They are easily discouraged when others cannot recognize their value.
- *Enjoyment type*. The enjoyment type is happy when engaged in culture, art, and the present, as opposed to the past or the future. Emotional well-being, self-enrichment, and quality of life are important rather than external values, such as competition and success. Therefore, they pursue a life of leisure full of experiences, such as travel, culture, and hobbies. They feel unhappy when they are tied to social norms, such as work and marriage. They are unhappy when time pressures are forced upon them from external sources.
- *Material success type*. The material success type feels happy when pursuing a family-centered life and working hard for material success. They are satisfied when living a well-organized life that matches the standards of success that social norms provide. They feel unhappy when their income is not constant, or their job is unstable, or when they do not meet expectations. Their level of self-esteem, in particular, depends on their possession of money. They are not interested in caring for others, or protecting the weak, or dedicating themselves to the public good. Even if they obtain as much material wealth as they expected, they are likely to feel empty and unfulfilled.
- *Parenting type*. The parenting type is happy when their children grow up in accordance with their expectations and receive praise from others. The most important concern in their life is childcare. When they have a good relationship with their children, they feel that everything is going well. Meanwhile, because their obsession with controlling their children is strong, it is easy to experience psychological conflicts when their children grow up and are beyond their control.

### 3.3. Distributions and Demographic Characteristics of the Seven Types of Happiness

We compared the distribution of the happiness types in the results of the Q methodology and the survey according to the calculation method presented above. Table 2 shows the distributions of the seven types of happiness analyzed by Q methodology and survey method.

The distribution of the happiness types from the survey result varies a lot from the Q analysis result. According to Danielson (2009), this distributional difference is a common phenomenon when the Q methodology and the survey are combined. Because the sampling method of Q methodology (P sample) follows a convenience sampling for a small number of people, it cannot represent the distribution of the entire population.

Instead, the purpose of Q methodology is to extract all happy or unhappy experiences (Q statements) from a small group of people and to ascertain the existing kinds of happiness patterns. Thus, a process of generalizing the identified types to the entire population is necessary. We performed a survey for the purposes of verifying and generalizing the types. By performing the generalization, we recognized the types and distributions of happiness that represent the Korean population.

We compared the differences in basic demographic characteristics, such as age, sex, education level, marital status, and number of children in the seven types of happiness. Table 3 summarizes the sociodemographic characteristics (mean value and percentages) of each happiness type. Differences in all demographic characteristics between each type were statistically significant.

The mean value of the age of each happiness type was different for each of the seven types (ANOVA (analysis of variance), *p* < 0.001). Type 2 (Belongingness) was the youngest, whereas Type 5 (Enjoyment) was the oldest. For the distribution of sex (*χ*^2^ analysis, *p* < 0.05), there were more males (63.6%) in Type 4 (Social recognition), and more females in Type 5 (Enjoyment). On education level (*χ*^2^ analysis, *p* < 0.01), people in Type 2 (Belongingness) had the highest education level (70.8% were college graduates or higher), whereas Type 7 (Parenting) had the lowest (37.6% were college graduates or higher). For the marital status (*χ*^2^ analysis, *p* < 0.001), more people were married in Type 3 (Mission), Type 4 (Social recognition), Type 5 (Enjoyment), Type 6 (Material success), and Type 7 (Parenting), but only half of the people were married in Type 1 (Self-actualization). Notably, only 25% were married in Type 2 (Belongingness).

### 3.4. Perceptions of Household Income and Overall Happiness of the Seven Types of Happiness

Perceived household incomes were different among the groups (ANOVA, *p* < 0.001). People in Type 6 (Material success; mean value, 5.05) and Type 7 (Parenting; mean value, 4.77) showed the two lowest values. Meanwhile, although not statistically significant (*p* = 0.19), people in Type 6 (Material success) were the highest earners compared with those in the other groups (Table 4). 

**Figure 1:**
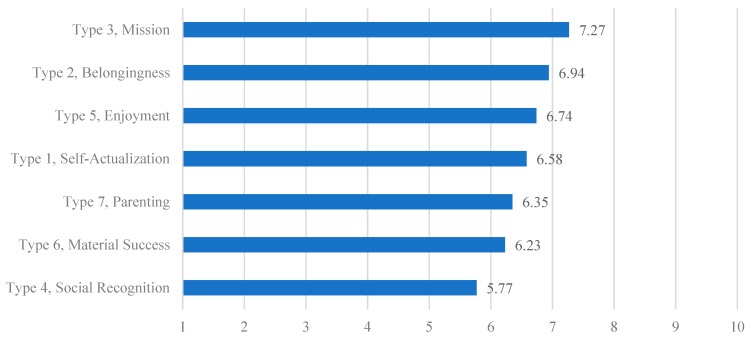
Seven happiness types and their perceived overall happiness level.

As shown in Figure 1, there was also a significant difference in perceived overall happiness (*p* < 0.001) among the seven types. Perceived overall happiness was the highest in Type 3 (Mission; mean value, 7.27) and lowest in Type 1 (Social recognition; mean value, 5.77). People in Type 6 (Material success; mean value, 6.23) and Type 7 (Parenting; mean value, 6.35) reported low perceived overall happiness. People in Type 2 (Belongingness; mean value, 6.94), Type 5 (Enjoyment; mean value, 6.74), and Type 1 (Self-actualization; mean value, 6.58) expressed relatively higher levels of perceived overall happiness.

## 4. Discussion

The study on the subjectivities of happiness is an attempt to understand the layperson’s different conceptions of happiness. People usually do not know why and when in their daily lives, they are happy or unhappy. In particular, many Koreans, at times, substitute money for happiness and undervalue their own value and the value of their families and their communities. Happiness should not be treated as an object to pursue unconditionally, but to be understood and learned systematically. As the happiness that people seek in a society may differ from one to another, it is necessary to present opportunities to identify with the various types of happiness suggested in this study.

More interesting and paradoxical results emerged in the comparison between happiness types in the survey data. When Koreans think of happiness, they, in fact, tend to value money the most. Of course, age and education are also important, but all of them are closely related to money. Therefore, Koreans regard money as the best solution for obtaining happiness. However, people who pursue money (Type 6, Material success) or social success (Type 4, Social recognition) feel the unhappiest. In particular, the material success type scored higher than the other types in the level of estimated total monthly household income, but they subjectively felt that their perceived household income was relatively low. Conversely, the Mission Type was not different from the Material Success Type in the level of estimated total monthly household income, but they subjectively felt their perceived household income was relatively high. In addition, the happiness level of the Mission Type was the highest among all the types. When we observed the variable of money as an objective measure in a decontextualized situation, it seemed that happiness grew systematically with more money. However, when we reversed the picture to look at subjectivities, including life context, material success, and social recognition, the money seeker’s happiness was revealed to be the lowest. These paradoxical results are consistent with previous studies explaining that not only Korean adults but also many people around the world pursue happiness in the wrong way. In fact, individuals who excessively select material goals as criteria for happiness experience long-term impairment in life satisfaction, while in the long run, seeking happiness in a socially engaged way would increase their life satisfaction [30,31,32].

Our study has some implications in terms of theoretical, methodological, and practical aspects. First, in relation to lay conceptions of happiness studies [10,11,12,33], we suggested a taxonomic basis for the seven types of happiness. We successfully captured the complexity of adult happiness in Korea and specifically illustrated the layperson’s diverse viewpoints on happiness in everyday life.

Second, we took a person-centric approach instead of a variable-centric one as a research method. Person-centric approaches regard the individual as the unit of analysis and posit that different types of happiness may change among individuals across situations and over time [8,34,35]. Through the Q methodology, we attempted to overcome the limited Western cultural norms and widen our understanding of the ecological structure of the concept to construct diverse conceptions of happiness in Korean society.

Third, the typology of happiness can provide useful guidelines for personalized intervention to increase happiness in individuals or establishing strategies for policymaking to promote public happiness. To increase the effectiveness of individual intervention or policymaking, it will be necessary to develop customized strategies on different types of happiness [36]. Each type of happiness needs different forms of support, resources, and opportunities to enhance happiness. The better the understanding of the preferable modes of happiness, the better the strategies that can be devised and delivered to the correct people [37]. On this point, our results yield practical knowledge about defining individual happiness and nudging environmental and ecological contexts to support the enhancement of happiness in Korean society. 

Further research will be needed to validate the possibility of a generalization of that model to ascertain whether it would be valid for different countries or cultures. Based on our typology and model, the development of brief and practical measurements will be helpful to evaluate what types of happiness people prefer and to design appropriate education and intervention programs to enhance happiness for each type through additional research. Longitudinal analyses about the stability and changes in types of happiness will be needed to explore the adult life path.

## 5. Conclusions

Using mixed methods combining personal in-depth interviews, focus group interviews, Q methodology, and the survey method, we examined four questions: (a) identifying the subjective adult happiness types in Korea, (b) describing the behavioral characteristics of a happy and an unhappy life as perceived by each type, (c) verifying the subjective types statistically, and (d) comparing the perceived household income and perceived overall happiness by type.

In analyzing the results, we found that the main types of happiness in Korean adults comprise a seven-factor structure: (1) Self-actualization, (2) Belongingness, (3) Mission, (4) Social recognition, (5) Enjoyment, (6) Material success, and (7) Parenting. These results reveal seven important value structures of happiness, which can capture the entire dynamic of adult happiness in Korea, along with diversity and changing properties across the course of an adult’s life.

We attempted to understand the complexity of happiness in adulthood through in-depth interviews, focus group interviews, and Q methodology, instead of taking a comparative approach of measuring happiness as a quantitative indicator among countries [38]. Using these mixed methods that included qualitative and quantitative approaches, we could shed light on the individual differences and shared commonalities of diverse conceptions of happiness. Our results provide useful guidelines for personalized intervention to increase happiness in individuals or for policymakers to promote public happiness. To enhance each type of happiness, different forms of support, resources, and opportunities are needed. The better the understanding of preferable modes of happiness, the better the strategies that can be devised. In addition, the development of a happiness type scale can be helpful in evaluating the type of happiness with which people identify and in designing the appropriate education or intervention programs to enhance happiness for each type.

## Figures and Tables

**Table 1:** Eigenvalue and total variance of the seven types of happiness.

	Factor 1	Factor 2	Factor 3	Factor 4	Factor 5	Factor 6	Factor 7
Eigenvalue	28.47	3.95	2.04	1.88	1.59	1.56	1.50
Total variance (%)	45.18	6.28	3.23	2.98	2.52	2.47	2.38
Cumulative variance (%)	45.18	51.46	54.69	57.67	60.19	62.67	65.05
Frame of reference	Fun of work	Sense of belonging	Achievement of mission	Social status	Leisurely life	Material success	Child rearing
Type of happiness	Self-actualization	Belongingness	Mission	Social recognition	Enjoyment	Material success	Parenting

**Table 2:** Distributions of the seven types of happiness (Q methodology and survey analysis results).

Types of Happiness	Result of Q Methodology		Result of Survey	
	Frequency	Percent	Frequency	Percent
Type 1, Self-actualization	36	57.1	301	30.1
Type 2, Belongingness	5	7.9	144	14.4
Type 3, Mission	4	6.3	131	13.1
Type 4, Social recognition	6	9.5	44	4.4
Type 5, Enjoyment	3	4.8	105	10.5
Type 6, Material success	3	4.8	165	16.5
Type 7, Parenting	6	9.5	109	10.9
Sum	63	100.0	999	100.0

**Table 3:** Demographic characteristics of the seven types of happiness.

Types of Happiness	Age ***	Sex *		Education Level **			Marital Status ***		Number of Children ***
		Male	Female	Less than High School	High School Graduate	College Graduate or Higher	Single	Married	
Type 1 Self-actualization	40.09	50.2%	49.8%	14.0%	30.9%	55.1%	49.5%	50.5%	1.15
Type 2 Belongingness	33.58	57.6%	42.4%	4.9%	24.3%	70.8%	75%	25.0%	.60
Type 3 Mission	47.01	42.7%	57.3%	14.5%	32.1%	53.4%	0%	100%	1.90
Type 4 Social recognition	43.32	63.6%	36.4%	15.9%	22.7%	61.4%	38.6%	61.4%	1.27
Type 5 Enjoyment	51.22	39.0%	61.0%	21.9%	40.0%	38.1%	0%	100%	2.30
Type 6 Material success	49.36	52.7%	47.3%	12.1%	41.2%	46.7%	16.4%	83.6%	2.07
Type 7 Parenting	50.59	45.0%	55.0%	17.4%	45.0%	37.6%	2.8%	97.2%	2.20
Total	44.05	49.5%	50.5%	13.7%	33.9%	52.4%	30.4%	69.6%	1.56

* *p* < 0.05, ** *p* < 0.01, *** *p* < 0.001.

**Table 4:** Seven types of happiness and perceptions of household income.

Types of Happiness	Perceived Household Income ***	Monthly Household Real Income (KRW Million)
Type 1 Self-actualization	5.38	239.68
Type 2 Belongingness	5.67	234.53
Type 3 Mission	5.71	265.63
Type 4 Social recognition	5.16	218.33
Type 5 Enjoyment	5.43	249.13
Type 6 Material success	5.05	268.16
Type 7 Parenting	4.77	231.13
Total	5.34	246.88

*** *p* < 0.001.

## Appendix A

**Table A1:** List of 93 Q statements.

Category	Q Statements	Likert Q ^a^	Z-Scores of Each Happiness Type (|Z| > 1.0) ^b^						
			1	2	3	4	5	6	7
Career	1. My childhood dream came true.	U					−1.2		
	2. It is not a life I dreamed, but I am doing what I like to do.	H					1.2		1.5
	3. I have never had any dreams.	U	−1.8		−1.9	−1.3	−1.4	−1.7	
	4. I always try to find out what my dream is.	H			1.1				
	5. I work harder than others.	H			1.6				
	6. I freely do what I want to do, whenever.	H	1.1			1.6			
	7. I commute at a fixed time.	H						1.3	
	8. My expertise at what I do is growing.	H, U	1.1	−1.1					
	9. I learn new things constantly.	H, U	1.2		1.3	1.3		−1.1	
	10. I challenge myself to solve difficult problems.	H					1.7		
	11. I am a pioneer in a particular area.								
	12. I adhere to a daily routine.	U	−1.4						
	13. I changed majors or careers and began a new one.								
	14. I challenge myself to try again after failures.	U							−1.2
	15. I do things that are helpful to others	H	1.0		1.0		1.2		
	16. I contribute to the public good.	U						−1.2	
	17. I have fun doing my work.	H	1.8						
	18. I am diligent and live in harmony with others.	H		1.6	1.0			1.9	1.7
	19. I work with various people.	H					1.3	1.3	
	20. I work hard to be rich.	U	−1.1						
	21. I am recognized in my job.	H	1.2	1.8	1.2	1.8			
	22. I feel that my work is important and valuable.	H	1.7		1.5	1.2			
	23. My work is suitable for my true self.	H, U	1.4				1.4	−1.4	
	24. My social status is getting noticeably higher.	H, U		−1.3		1.0			−1.0
	25. I have a clear purpose in what I do.	H			2.1	1.9			
	26. I do not know what to do next.	U	−1.7			−1.5			
	27. I take the initiative at work.								
	28. I do not have the right to make decisions on what I do.	U	−1.8		−1.1	−1.9	−1.7	−1.4	−1.0
	29. People around me follow my words.	H							1.0
	30. I have an unstable job.	U	−1.5	−1.9		−1.6		−2.1	−1.9
Community	31. I work in a community with people of similar values.	H	1.1						
	32. We are free to do what we want to do in our community.								
	33. I run a community with a clear purpose and direction.								
	34. I actively participate in online communities.	U		−2.0					
	35. I chat anonymously on the internet and share words of comfort.	U		−1.9	−1.2	−1.2	−1.3		−2.1
	36. My meetings with people in online communities extend to offline meetings.	U		−1.9	−1.4	−1.4			−1.3
Finances	37 I am insecure because my income is unstable.	U	−1.7			−1.4	−1.7	−2.4	−1.5
	38. I only need a minimum amount of money.	H		2.0			1.2		
	39. Money, cars, stocks, and houses are frequent topics in my conversations with others.	H, U	−1.4		−1.8			1.2	
	40. Anytime I decide, I can earn money.	H, U				1.4		−1.1	
Health	41. I live a well-regulated life for good health.	H		1.4		1.3		2.1	
	42. I am sick.	U	−1.8	−1.3	−1.5	−2.1	−1.8		−1.7
	43. I have given birth to a child.	H			1.5			1.4	2.6
	44. As I grew older, my appearance changed.								
	45. After I got sick, I began to take care of the people around me.	H, U					−1.5	1.2	
Interpersonal Relationship	46. I have someone to talk to when I am in trouble.	H	1.3	1.4	1.5	1.3			1.7
	47. I try to satisfy other people’s expectations.	H			1.3				
	48. I am willing to help someone in a difficult situation.	H		1.8					
	49. I focus on what I want to do without worrying about other people’s gaze.	H		1.2	1.3				
	50. I interact extensively with various people.	H				1.1	1.3		
	51. I have to do better than others.	U	−1.4						
	52. I have a boss who serves as a mentor.	U		−1.2					
	53. I have subordinates at work who are more competent than me.	U		−1.0	−1.5	−1.4		−1.1	−1.3
	54. Loving others helped me grow.								
	55. I have a friend who shares my heart.	H	1.7	1.1		1.8	1.5	1.3	1.6
	56. Married couples help each other in difficult situations.	H, U	1.2		−1.7	1.1	1.3		1.9
	57. My spouse is socially recognized.	U			−1.3				
	58. My spouse resolves most of my difficulties.	U		−1.0	−1.7		−1.6		
	59. Even if we are a married couple, we handle our own problems individually.	H, U					−2.1		1.3
	60. I experience emotional discord with my spouse or in-laws.	U	−1.9	−1.1	−1.3	−2.1	−2.0	−1.7	−1.6
	61. I must be married.	H, U		−1.0	−1.4			1.7	1.3
	62. I trust my children and support them.	H			1.2		1.7	2.2	1.8
	63. People around me praise my children.	H					1.8	1.0	1.9
	64. Because I am busy, our children are raised by others.	U	−1.1	−1.3	−1.3	−1.1			−1.5
	65. I have conflicts with my adolescent children.	U	−1.3	−1.0		−1.5	−1.8		−1.7
	66. I look for alternative child education methods.								
	67. Private tutoring is essential in my children’s education.	H, U	−1.2			−1.1		1.0	
	68. I have parents or family members who are struggling with illness.	U	−1.9	−1.7	−1.3	−2.1	−1.1	−1.3	−1.8
	69. I have conflicts with my family members.	U	−2.1	−1.3		−1.9		−1.4	
	70. I compare myself with others.	U	−1.8			−1.8		−1.4	
	71. I cannot build good relationships with others because I am busy.	U	−1.4			−1.5	−1.3	−1.3	
Leisure	72. I enjoy cultural and artistic hobbies.	H, U	1.1				1.9	−1.3	
	73. I communicate with close friends regularly.	H, U			−1.2		1.3	1.4	
	74. I spend time in nature whenever I can.	H		1.3			1.4	1.9	
	75. I drink and go to a karaoke place to relax.	U		−1.0	−1.1			−1.5	−1.3
	76. My hobby is exercising.	H, U		1.1		1.1		1.5	−1.2
	77. On days off, I spend my time watching TV, reading books, surfing the internet, etc.								
	78. I travel on weekends.	H, U		1.1	−1.2		1.9		
	79. I participate in community festivals with local residents during my leisure time.								
	80. Even though I am on vacation, I attend to work.	U	−1.5	−1.2		−1.4	−1.4		
Aging, Death, and Religion	81. The present is more important than the past or future.	H		1.6			1.2	1.2	1.5
	82. I am aware of my limitations as a human being and have a mind toward a higher being.	H			1.9			1.2	
	83. I regularly participate in religious activities.								
	84. I prepare for aging, such as ensuring my pension.	H				1.1			
Residential Environment	85. I live in a big city with good public transport.	H		1.1					1.4
	86. I live in a place where my mind is relaxed and nature is beautiful.	H, U	1.2	1.2			−1.1		
	87. I live in my hometown.	U		−1.2					
Stress Coping.	88. I perceive difficulties as a life process.	H		1.6	1.6				
	89. I approach my life with confidence.	H	1.7	1.8	1.2	1.3			
	90. I appreciate even the minor things.	H	1.2	2.0	1.8				1.5
	91. When I face a difficult situation, I try to solve the problem actively.	H		1.6			1.0	1.6	1.2
	92. When I am stressed, I listen to music or watch movies.								
	93. I try not to think about stress when I have a hard time.	U	−1.2				−1.8		

^a^ The Q statements were used as items in the questionnaire survey and scored using a Likert scale. “H” means the statement was used as a happiness items, and “U” means it was used as an unhappiness item. “H, U” means it was used as both happiness and unhappiness items. ^b^ The happiness type are as follows: 1 = Self-Actualization Type, 2 = Belongingness Type, 3 = Mission Type, 4 = Social Recognition Type, 5 = Enjoyment Type, 6 = Material Success Type, 7 = Parenting Type. Each Q statement has its own Z-score; only values higher than 1.0 are displayed.

## References

[1] World Bank GDP Per Capita (Current US$). https://data.worldbank.org/indicator/NY.GDP.PCAP.CD.

[2] Helliwell J.F., Layard R., Sachs J.D. (2019). World Happiness Report 2019.

[3] O’Brien C. (2013). Happiness and sustainability together at last! Sustainable happiness. Can. J. Educ..

[4] O’Brien C. (2012). Sustainable happiness and well-being: Future directions for positive psychology. Psychology.

[5] Walker H., Kavedžija I. (2015). Values of happiness. HAU J. Ethnogr. Theory.

[6] De Guzman A.B., Silva K.E.M., Silvestre J.Q., Simbillo J.G.P., Simpauco J.J.L., Sinugbuhan R.J.P., Sison D.M.N., Siy M.R.C. (2011). For your eyes only: A Q-Methodology on the ontology of happiness among chronically ill Filipino elderly in a penal institution. J. Happiness Stud..

[7] Gasper D. (2010). Understanding the diversity of conceptions of well-being and quality of life. J. Socio Econ..

[8] Wolf A. (2013). Wellbeing for public policy: Roles for Q Methodology. Operant Subj..

[9] Shim H.W. (2017). A study of subjectivity about happiness among undergraduate students: Q methodological approach. J. Korean Acad. Soc. Nurs. Educ..

[10] Bojanowska A., Zalewska A.M. (2016). Lay understanding of happiness and the experience of well-being: Are some conceptions of happiness more beneficial than others?. J. Happiness Stud..

[11] Hitokoto H., Takahashi Y., Kaewpijit J. (2014). Happiness in Thailand: Variation between urban and rural regions. Psychologia.

[12] Pramanik S., Ray D. (2018). Subjective construal of happiness among urban educated Bengali youth: A preliminary study using grounded theory approach. Indian J. Posit. Psychol..

[13] Lykken D. (2000). Happiness: The Nature and Nurture of Joy and Contentment.

[14] Lykken D., Tellegen A. (1996). Happiness is a stochastic phenomenon. Psychol. Sci..

[15] Erikson E.H. (1950). Childhood and Society.

[16] Joshanloo M., Rizwan M., Khilji I.A., Ferreira M.C., Poon W.-C., Sundaram S., Ho L.S., Yeung V.W.-I., Han G., Bae J. (2016). Conceptions of happiness and life satisfaction: An exploratory study in 14 national groups. Personal. Individ. Differ..

[17] Sagiv L., Roccas S., Oppenheim-Weller S., Joseph S. (2015). Values and well-being. Positive Psychology in Practice: Promoting Human Flourishing in Work, Health, Education, and Everyday Life.

[18] Sagiv L., Schwartz S.H. (2000). Value priorities and subjective well-being: Direct relations and congruity effects. Eur. J. Soc. Psychol..

[19] Levinson D.J. (1986). A conception of adult development. Am. Psychol..

[20] Kim M.S., Kim H.W., Cha K.H., Lim J. (2007). What makes Koreans happy: Exploration on the structure of happy life among Korean adults. Soc. Indic. Res..

[21] Yu N., Jeong Y., Kim B., Chong Y., Shin H. (2015). An exploratory study on the concept of happiness in Korean undergraduate students. J. Koreanol..

[22] Goodman F.R., Disabato D.J., Kashdan T.B., Kauffman S.B. (2018). Measuring well-being: A comparison of subjective well-being and PERMA. J. Posit. Psychol..

[23] Kim Y.-S. (2015). Perception of happiness among health-related university students: Q methodological approach. Indian J. Sci. Technol..

[24] Ryu C.S. (2016). Happiness of kindergarten teachers: A Q-methodology approach. J. Korea Acad. Ind. Coop. Soc..

[25] Creswell J.W. (2014). Research Design: Qualitative, Quantitative, and Mixed Methods Approaches.

[26] Brown S.R. (1980). Political subjectivity.

[27] Danielson S. (2009). Q method and surveys: Three ways to combine Q and R. Field Methods.

[28] Van Tubergen N. (1975). QUANAL User’s Guide (Computer Program Manual).

[29] Zabala A., Pascual U. (2016). Bootstrapping Q methodology to improve the understanding of human perspectives. PLoS ONE.

[30] Mogilner C. (2010). The pursuit of happiness:Time, money, and social connection. Psychol. Sci..

[31] Ford B.Q., Dmitrieva J.O., Heller D., Chentsova-Dutton Y., Grossmann I., Tamir M., Uchida Y., Koopmann-Holm B., Floerke V.A., Uhrig M. (2015). Culture shapes whether the pursuit of happiness predicts higher or lower well-being. J. Exp. Psychol. Gen..

[32] Rohrer J.M., Richter D., Brummer M., Wagner G.G., Schmukle S.C. (2018). Successfully striving for happiness: Socially engaged pursuits predict increases in life satisfaction. Psychol. Sci..

[33] Delle Fave A., Brdar I., Wissing M.P., Araujo U., Castro Solano A., Freire T., Hernández-Pozo M.D.R., Jose P., Martos T., Nafstad H.E. (2016). Lay definitions of happiness across nations: The primacy of inner harmony and relational connectedness. Front. Psychol..

[34] Busseri M.A., Sadava S., Molnar D., DeCourville N. (2009). A person-centered approach to subjective well-being. J. Happiness Stud..

[35] Shmotkin D. (2005). Happiness in the face of adversity: Reformulating the dynamic and modular bases of subjective well-being. Rev. Gen. Psychol..

[36] Cantor N., Sanderson C.A., Kahneman D., Diener E., Schwarz N. (2003). Life task participation and well-being: The importance of taking part in daily life. Well-Being: Foundations of Hedonic Psychology.

[37] Easterlin R. Building a better theory of well-being. Proceedings of the 2003 Conference “Paradoxes of Happiness in Economics”, University of Milano-Bicocca.

[38] Mathews G. (2012). Happiness, culture, and context. Int. J. Wellbeing.

